# Supplementary material for: Beyond Nitrogen in the Oxygen Reduction Reaction on Nitrogen-Doped Carbons: A NEXAFS Investigation
Source: Nanomaterials (Basel). 2021 May 1;11(5):1198. doi: 10.3390/nano11051198 (PMC8147284; doi:10.3390/nano11051198)
Supplement: Supplementary file 1 [file nanomaterials-11-01198-s001.zip › nanomaterials-1210923-supplementary.pdf]

Supplementary Material

Beyond Nitrogen in the Oxygen Reduction Reaction on Nitrogen-Doped Carbons: A NEXAFS Investigation

Eugenia Tanasa [1], Florentina Iuliana Maxim [2], Tugce Erniyazov [2], Matei-Tom Iacob [2], Tomáš Skála [3], Liviu Cristian Tanase [4], Cătălin Ianăşi[5], Cristina Moisescu [6], Cristina Miron [7], Ioan Ardelean [6], Vlad-Andrei Antohe [7,8],
Eugenia Fagadar-Cosma [5] and Serban N. Stamatin [2,7,*]

[1] Faculty of Applied Chemistry and Materials Science, Politehnica University of Bucharest, Splaiul Independentei Str. No. 313, 060042 Bucharest, Romania; eugenia.tanasa@physics.pub.ro
[2] Nano-SAE Research Centre, University of Bucharest, Atomistilor 405, 077125 Magurele, Ilfov, Romania; iuliana.maxim@3nanosae.org (I.M.); tugcegoktas95@gmail.com (T.G.); tom.iacob@3nanosae.org (T.I.)
[3] Department of Surface and Plasma Science, Charles University, V Holešovičkách 2, 18000 Prague,
Czech Republic; tomas.skala@elettra.eu
[4] National Institute of Materials Physics, Atomistilor 405A, 077125 Magurele, Ilfov, Romania; liviu.tanase@infim.ro
[5] "Coriolan Drăgulescu" Institute of Chemistry, Mihai Viteazul Ave. 24, 300223 Timisoara, Romania;
cianasic@yahoo.com (C.I.); efagadarcosma@acad-icht.tm.edu.ro (E.F.-C.)
[6] Department of Microbiology, Institute of Biology Bucharest, Splaiul Independenței 296, 060031, Bucharest,
Romania; cristina.moisescu@yahoo.com (C.M.); ioan.ardelean@ibiol.ro (I.I.A.)
[7] Faculty of Physics, University of Bucharest, Atomistilor 405, 077125 Magurele, Ilfov, Romania;
cristina.miron@fizica.unibuc.ro (C.M.); vlad.antohe@fizica.unibuc.ro (V.A.A.)
[8] Institute of Condensed Matter and Nanosciences (IMCN), Université catholique de Louvain (UCLouvain), Place Croix du Sud 1, B-1348 Louvain-la-Neuve, Belgium
* Correspondence: serban@3nanosae.org or serban.stamatin@unibuc.ro; Tel.: +40-214574838

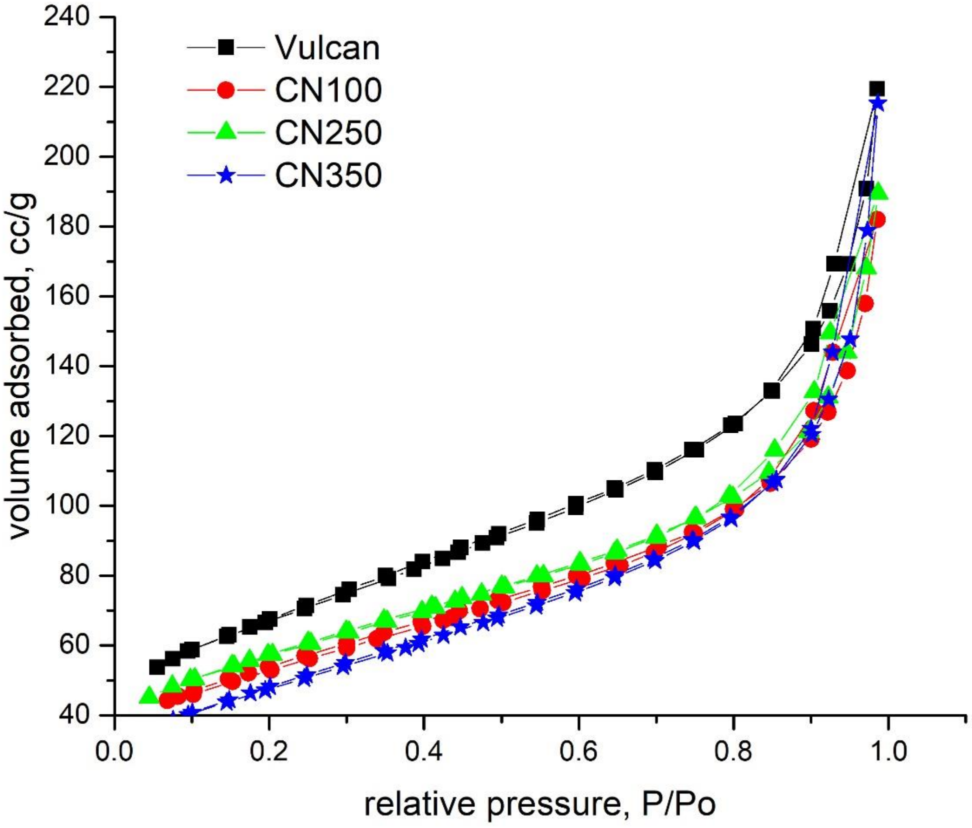

Figure S1. Nitrogen adsorbtion/desorbtion isotherms for Vulcan (black), CN100 (red), CN250 (green), CN350 (blue) samples.

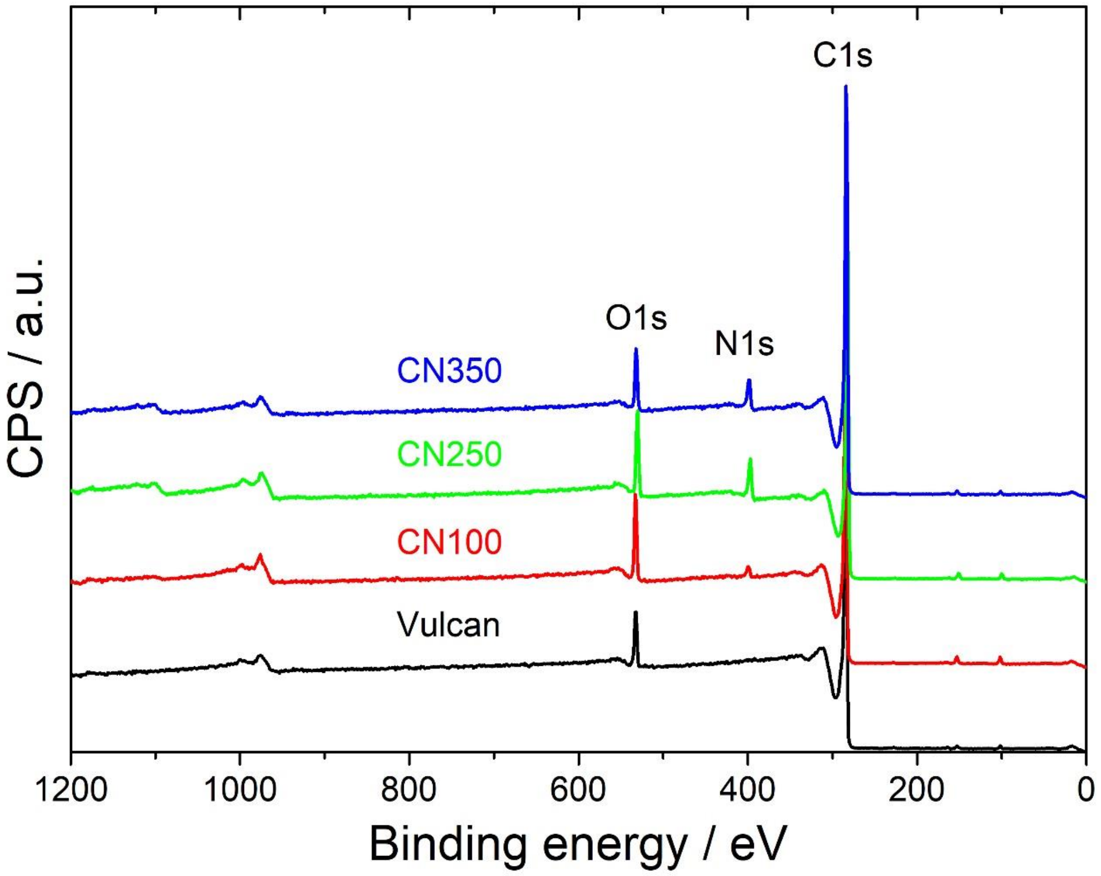

Figure S2. Wide spectrum X-ray photoelectron spectroscopy.

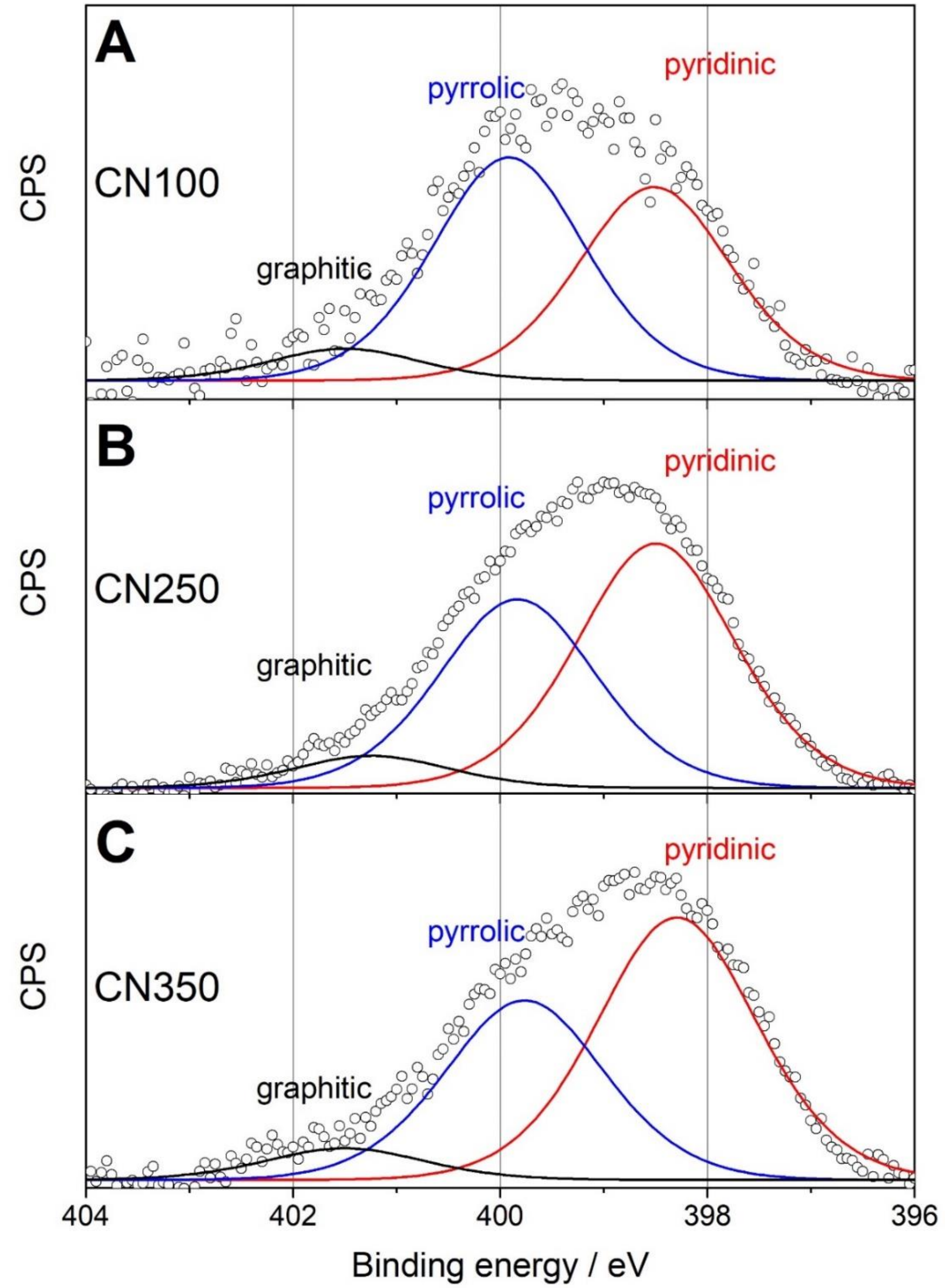

Figure S3. Deconvolution of the N 1s peak obtained from the synchrotron radiation photoelectron spectroscopy.

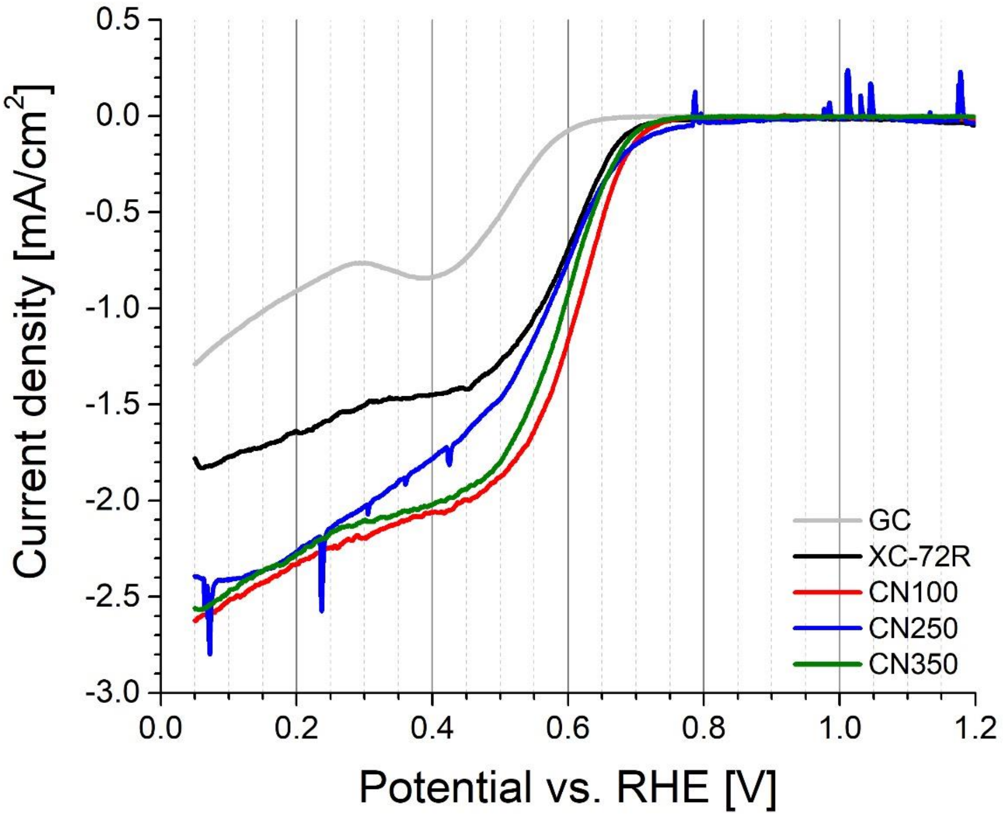

Figure S4. Oxygen reduction reaction curves obtained at 20 mV s-1 at 900 rpm in O2 saturated 0.1 M KOH.

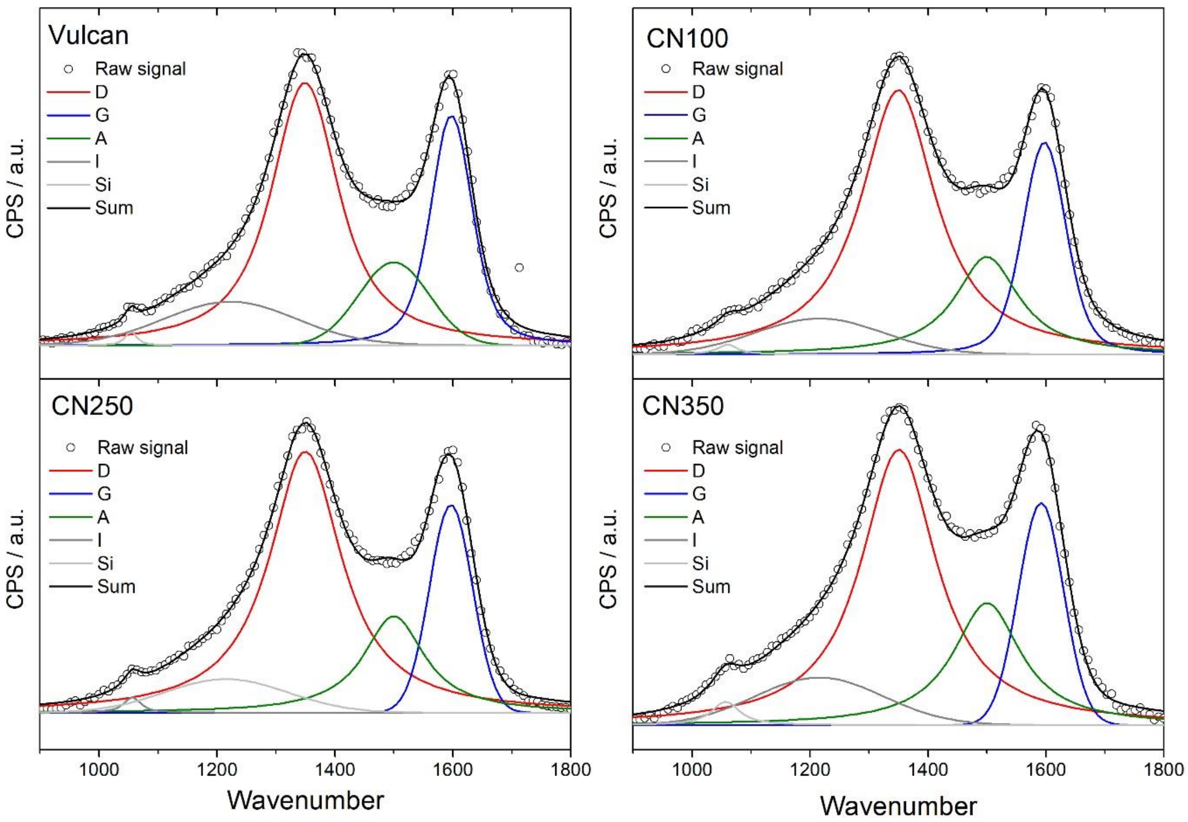

Figure S5. Raman peaks recorded for Vulcan, CN100, CN250 and CN350 with a 532 nm laser and a bandstop filter to remove the Rayleigh component. Deconvolution was done in Fityk with Voigt functions only. The A peak was locked at 1500 cm^{-1} while other peaks were allowed to shift by less than 5 cm^{-1}.

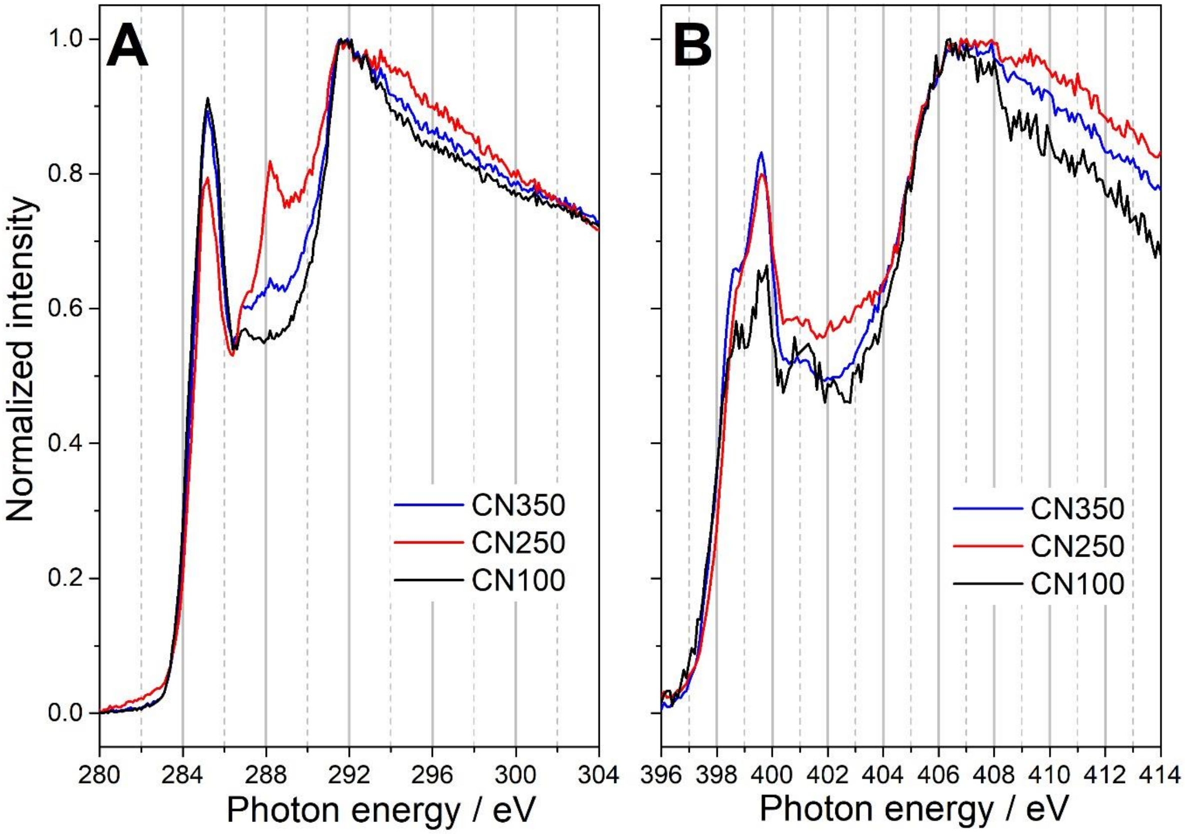

Figure S6. Near edge X-ray absorption fine structure of nitrogenated samples.

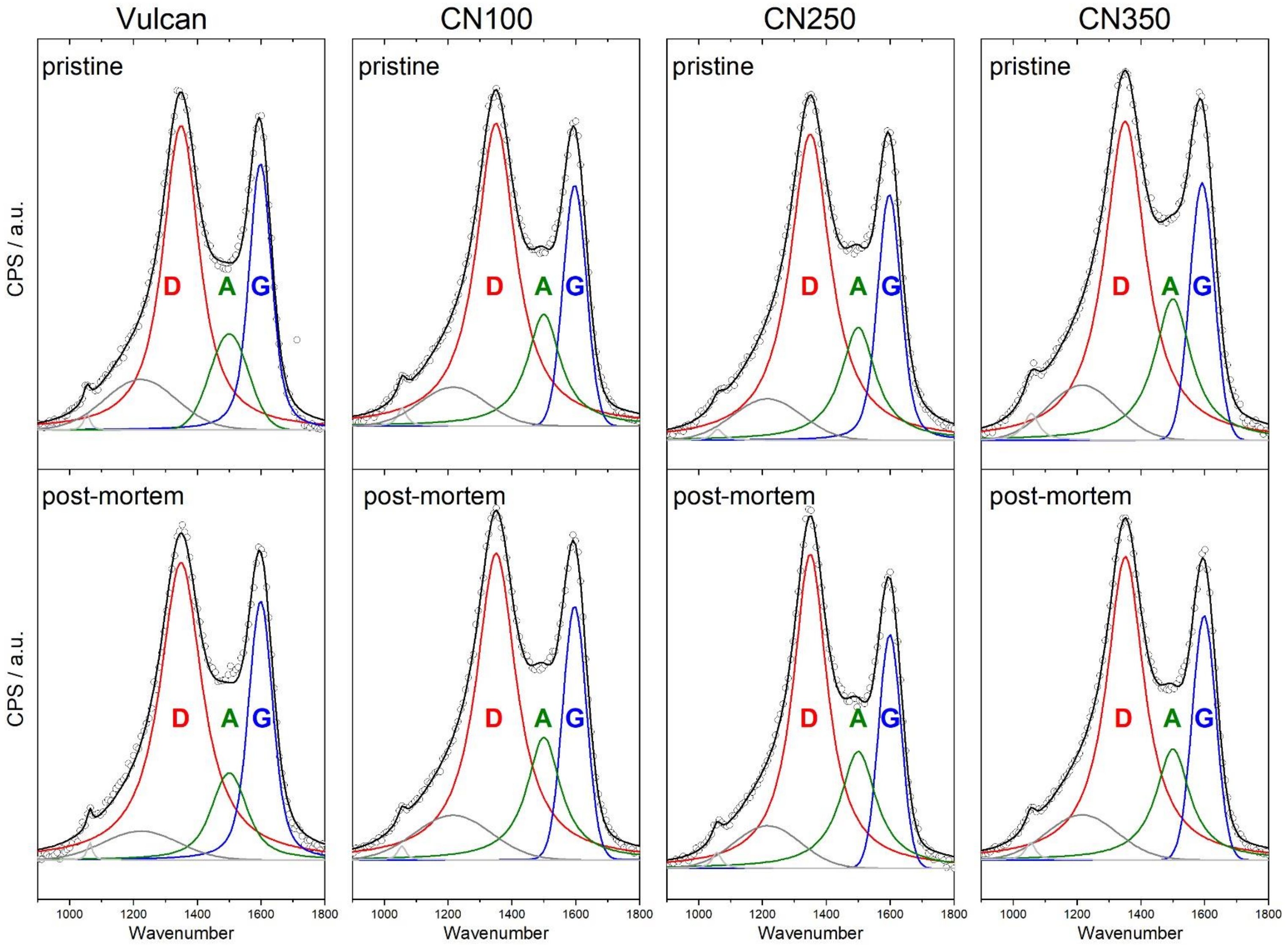

Figure S7. Raman spectra of pristine samples (top row) and post-mortem samples (bottom row). Pristine samples were identical to Figure S4. The decovolution procedure used in Figure S4 was applied here. Post-mortem samples were subjected to a 6 h accelerated stress test between 0.05 and 1.20 V vs. RHE in 0.1 M KOH.

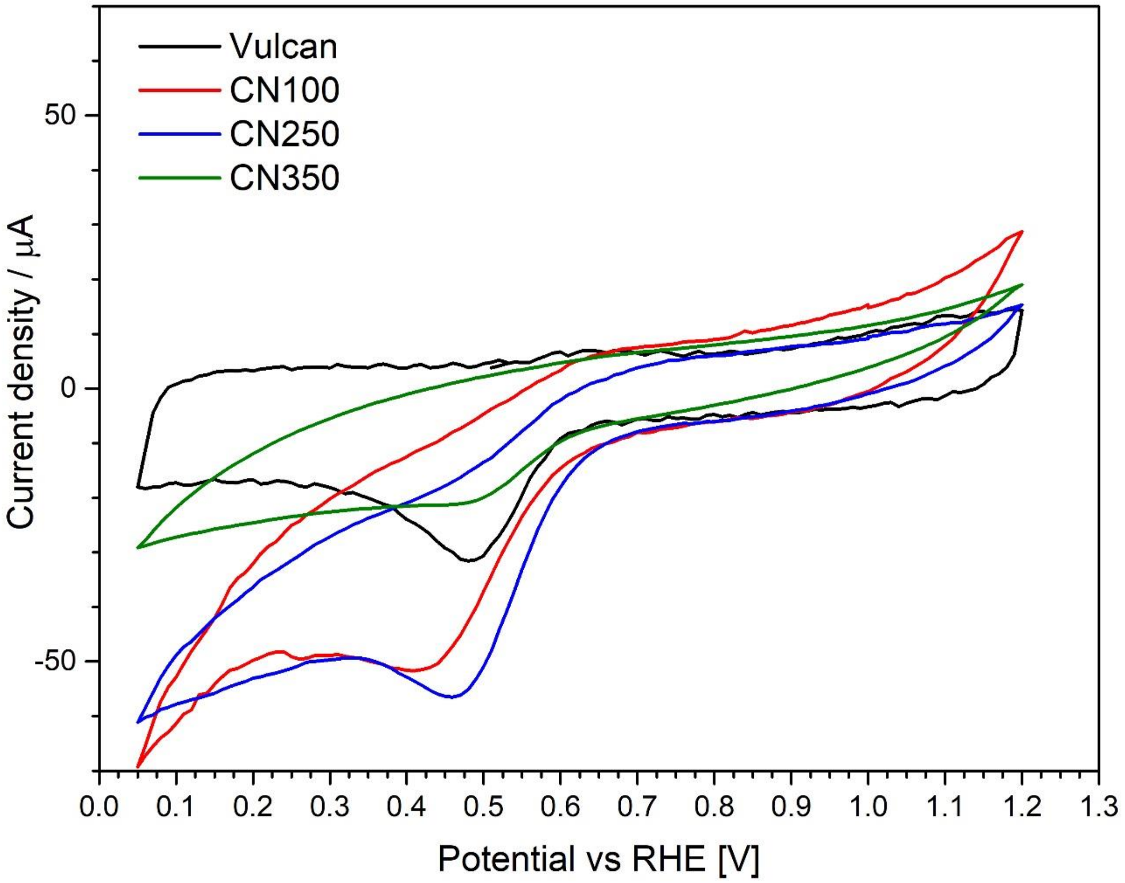

Figure S8. CVs at the end of the accelerated stress test (6 h scanned at 0.1 V s^{-1} between 0.05 and 1.20 V vs. RHE in 0.1 M KOH).
